# Search for Cellular Stress Biomarkers in Lymphocytes from Patients with Multiple Sclerosis: A Pilot Study

**DOI:** 10.1371/journal.pone.0044935

**Published:** 2012-09-13

**Authors:** Sabrina Grecchi, Giuliano Mazzini, Antonella Lisa, Marie-Therese Armentero, Roberto Bergamaschi, Alfredo Romani, Fabio Blandini, Carol Di Perri, Anna Ivana Scovassi

**Affiliations:** 1 IGM-CNR, Pavia, Italy; 2 IRCCS Istituto Neurologico Nazionale C. Mondino, Pavia, Italy; Klinikum rechts der Isar der Technischen Universitaet Muenchen, Germany

## Abstract

Multiple Sclerosis (MS) is a chronic disease of the central nervous system, the etiology of which, although not completely known, involves inflammation and autoimmunity. In the present study we aimed at identifying molecular markers of apoptosis, cellular stress and DNA damage in isolated peripheral blood mononuclear cells (PBMCs) of MS patients. The analysis was carried on 19 relapsing-remitting untreated MS patients and 13 healthy individuals. We investigated the emergency-driven synthesis of poly(ADP-ribose) (PAR), the expression level of the constitutive enzyme poly(ADP-ribose) polymerase-1 (PARP-1) and the DNA damage-induced phosphorylation of histone H2AX. PAR accumulation, PARP-1 and phosphorylated H2AX (γH2AX) were detected by immunofluorescence experiments on PBMCs isolated from 19 patients and 13 healthy volunteers. Our results show for the first time a net increased amount in PAR and γH2AX in MS patients compared to healthy individuals. Patients were further subdivided in three groups, according to the neuroimaging (MRI)-based classification of disease phase. Remarkably, we found a positive correlation between the level of γH2AX and MS aggressiveness. In addition, apoptosis in PBMCs was monitored by flow cytometry of both phosphatidylserine exposure (revealed by Annexin V-FITC labeling) and membrane permeability to propidium iodide. Our observations provide the evidence that the number of apoptotic cells was significantly higher in patients compared to healthy individuals, thus suggesting that apoptosis could affect MS lymphocyte function.

## Introduction

The etiology of Multiple Sclerosis (MS) is not known and probably implies a multifactorial context. Pathogenetic mechanisms of MS have been extensively investigated and imply loss of tolerance in the immune response [1], [2] and inflammatory aggression towards oligodendrocytes in the myelin sheath as well as neurodegenerative contributions [3].

Oxidative stress that produces Reactive Oxygen Species (ROS) harmful for cells, proteins and DNA, has been claimed to be involved in MS at the target tissue, within the Central Nervous System (CNS) [4]. However, similar effects may act at the inflammatory effector (*i.e.* lymphocyte) level, affecting the control of apoptosis, which has been also involved in MS pathogenesis [5].

In this study, we focused on markers of DNA damage and cellular stress by analysing respectively DNA double strand break (DSB)-induced serine-139 phosphorylation of histone H2AX (a widely used marker of DNA damage) [6], [7] and poly(ADP-ribose) accumulation, which is catalysed by poly(ADP-ribose) polymerases (PARPs) in response to cellular stress conditions [8]. Moreover, we evaluated the occurrence of apoptosis using flow cytometry. These investigations were conducted in peripheral blood mononuclear cells (PBMCs) from MS patients and control subjects, using samples collected on the same day. The association with the disease evolution and disease phase was explored assessing lesion load changes and presence of gadolinium (GD) enhancement in brain and spinal Magnetic Resonance Imaging (MRI).

The final aim of our study was to evaluate whether these indices of peripheral DNA damage and cellular stress may provide an innovative set of biomarkers of MS, which may be useful for follow-up monitoring.

**Figure 1:**
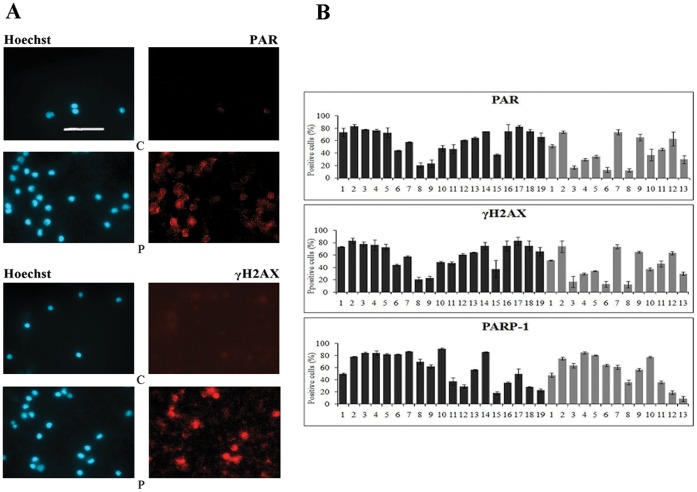
Analysis of PAR and γH2AX in PBMCs from healthy donors and MS patients. (A) Indirect immunofluorescence (IIF) analysis of cell morphology, visualised after Hoechst staining (blue fluorescence), PAR synthesis (upper panel) and γH2AX level (lower panel) (TRITC fluorochrome, red fluorescence). Representative results of C3 and P14 are shown. Scale bar: 50 µm. (B) Quantification of data from IIF. Black bars: MS patients (N = 19); grey bars: healthy donors (N = 13). Data are expressed as mean ± S.D. calculated from three independent experiments.

## Subjects and Methods

### Patients and Healthy Donors

We analysed freshly isolated peripheral blood mononuclear cells (PBMCs) from 19 patients with MS and from 13 healthy volunteers. MS patients were enrolled at the IRCCS Istituto Neurologico Nazionale C. Mondino, Pavia, Italy, on the basis of the following criteria: less than 5 years from the onset of disease, treatment-naïve, with Expanded Disability Status Scale (EDSS) ranging between 0 and 6, and regularly monitored by contrast-enhanced MRI (Magnetic Resonance Imaging). Demographic and clinical data of MS patients and healthy donors are summarized in Table 1. The study protocol was approved by the local ethical committee; before being enrolled, subjects participating in the study signed an informed consent form.

**Table 1:** Demographic and clinical data of patients with RRMS and healthy donors.

	PATIENTS	HEALTHY DONORS
*Number*	19	13
*Female/Male*	11/8	4/9
*Age (mean)*	42.6	42.8
*EDSS median (range)*	1.0 (0–4.5)	–
*MRI*	class 1 (unchanged) 10class 2 (GD^-^, increased lesion load) 4class 3 (GD^+^, GD enhancing lesion) 5	–
*Disease modifying therapies*	None	–

EDSS: Expanded Disability Status Scale; MRI: Magnetic Resonance Imaging. Class 1: unchanged. Class 2: Increased lesion load: at least one T2 lesion not present in the previous scan; GD^-^: no Gadolinium enhancing lesions. Class 3: GD^+^: at least one Gadolinium enhancing lesion.

**Figure 2:**
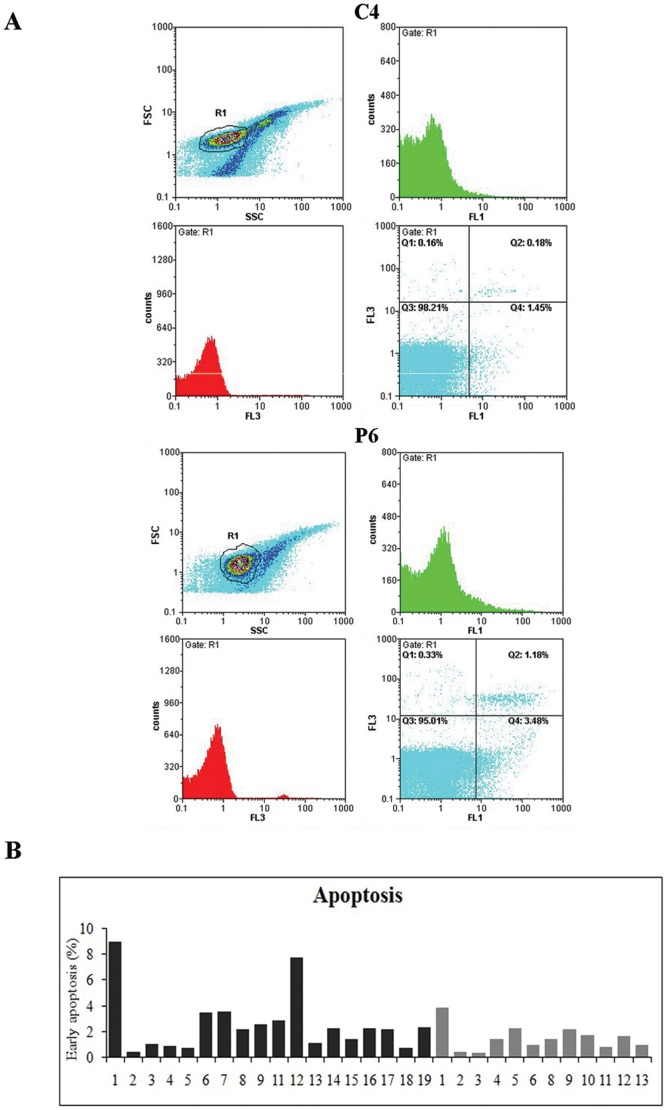
Apoptosis in PBMCs from healthy donors and MS patients. (A) Flow cytometry analysis of apoptosis in PBMCs from healthy donor C4 and MS patient P6 stained with propidium iodide (red fluorescence, FL3) and Annexin V (green fluorescence, FL1). Forward scatter (FSC) defines cell population morphology; double immunocytochemistry labeling (FL1 and FL3) identifies four subpopulations, *i.e*. alive (Q3), early apoptotic (Q4), late apoptotic (Q2) and necrotic (Q1) cells. The relative percentage of each subpopulation is shown. SSC: side scatter. (B) Quantification of data from flow cytometry analysis. Black bars: MS patients; grey bars: healthy donors. The values represent the percentage of early apoptotic cells in the whole population; the assay has been performed on 18 MS patients and 12 healthy individuals.

### Isolation of PBMCs from Peripheral Blood

PBMCs were obtained by centrifugation of whole blood (∼9 ml) through Ficoll (Sigma-Aldrich) at 2000 rpm for 20 min at room temperature; lymphocyte-monocyte fraction was taken, washed with PBS and centrifuged at 1100 rpm for 15 min at room temperature. Cellular pellets were resuspended in 5 ml of PBS (Phosphate Buffered Saline) and used to prepare about 30 coverslips (20×20 mm) with 20 µl of PBMC suspension for Immunocytochemistry experiments. Aliquots of about 5×10^5^ cells were used for flow cytometry; about 3×10^6^ cells were pelleted and kept in liquid nitrogen until further use.

**Figure 3:**
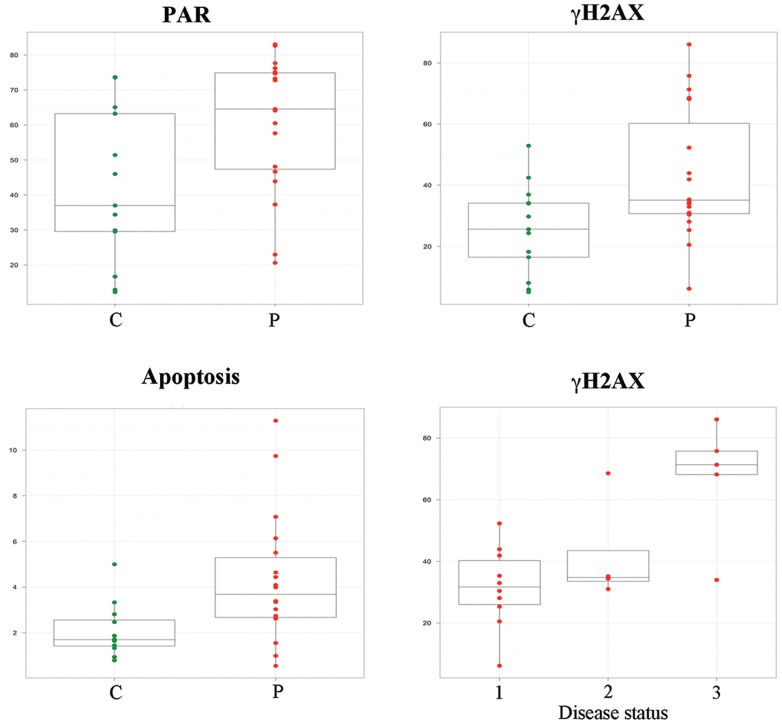
Correlation of biological data with disease. Box plots represent the distribution of PAR accumulation (upper left), γH2AX level (upper right) and apoptosis (lower left) in healthy subjects (C, green) and in patients (P, red). The lower right box plot shows the γH2AX distribution in the three classes of the disease status (according to the description in Subjects and Methods).

### Immunocytochemistry

PARP-1 expression, PAR synthesis and phosphorylation of histone H2AX (γH2AX) were analysed through Indirect ImmunoFluorescence (IIF).

For PARP-1, cells were fixed with 2% paraformaldehyde (PFA) for 10 min at room temperature, washed with PBS for 5 min, then incubated with 70% ethanol for 30 min or overnight at −20°C. Then, cells were rehydrated with PBS and incubated with PTN (PBS containing 10% newborn calf serum and 0.1% Tween-20) for 30 min at room temperature, with the monoclonal antibody C2–10 (Alexis Biochemicals) diluted 1∶200 in PBS for 1 h at room temperature. Cells were washed 5 times for 5 min with PBS, incubated for 30 min with the secondary anti-mouse IgG antibody conjugated with TRITC fluorochrome (Jackson ImmunoResearch Lab., diluted 1∶50 in PBS), washed 5 times for 5 min with PBS, stained with 0.1 µg/ml Hoechst 33258 for 10 min and finally washed twice for 10 min with PBS. Finally, coverslips were mounted with 30 µl of Antifade solution (90% glycerol, 20 mM Tris-HCl pH 7.5, 0.1% DABCO).

For PAR detection, cells were fixed with 2% PFA for 15 min in ice, washed 2 times for 5 min with cold PBS, then permeabilised with 0.1% Triton-X100 for 3 min in ice. After washings with PBS and incubation with PTN (as above), samples were incubated for 30 min at 37°C with the monoclonal antibody 10H (Alexis Biochemicals) diluted 1∶100 in PBS, and processed as previously described.

Serine-139 phosphorylation of H2AX was monitored by fixing the cells with cold methanol for 4 min on ice. Cells were incubated with the monoclonal antibody JBW301 (Millipore), diluted 1∶5000 in PBS containing 5% skim milk (Gene Spin), for 1 h at room temperature. Samples were then processed as above.

Each experiment for PAR and γH2AX analysis includes a negative control represented by untreated HeLa cells, and a positive one, constituted by cells treated with the DNA-damaging agent bleomycin under conditions leading to DNA damage and, by consequence, PAR synthesis. Three independent experiments were carried out.

Measurement of fluorescence intensity was obtained with an Olympus IX71 microscope equipped with a 60X objective. The images were acquired through a digital camera Cool SNAPES (PhotoMetrics) using the MetaMorph acquisition software; Adobe Photoshop was used as elaborating software. For each sample, 500 cells were analysed by two investigators. Positive cells were counted and data expressed as the number of positive cells over the total cell number in the same field.

### Flow Cytometry

For each subject an aliquot of about 5×10^5^ cells was processed with the “Annexin kit” (Bender Medical System) according to the manufacturer’s protocol. Briefly, cells were washed in PBS and the pellet resuspended with 100 µl of Binding Buffer, incubated for 15 min at room temperature; then, 5 µl of Annexin V-FITC (1 µg/µl) were added and cells kept for 15 min at room temperature with occasional shaking. Finally, 2 µl of propidium iodide (1 µg/ml) were added, and samples incubated for 10 min at room temperature in the dark. Samples were diluted with PBS to a final volume of 1500 µl and immediately analysed by means of a flow cytometer Partec PAS II (Munster Germany) equipped with an argon ion laser excitation of 20 mW at 488 nm. At least 50,000 events were collected from each measurement. Data were stored in the PC memory of the instrument, gated and elaborated according to the software “Flow-Max” of the PAS II.

### Magnetic Resonance Imaging (MRI)

Image Acquisition: in concomitance with blood sampling, patients were examined with a 1.5T Philips Intera Gyroscan 8-channel scanner. The following sequences were acquired for brain MRI: 2D multi-planar axial and sagittal fast Fluid Attenuated Inversion Recovery (FLAIR) T2-weighted image (WI); 3D high resolution (HIRES) T1-WI using a fast gradient echo (FFE). For cervical and thoracic spinal MRI, the following sequences were used: sagittal turbo spin echo (TSE)-T2 and sagittal TSE-T1. All patients were examined after *i.v.* gadolinium (GD) injection (0.4 ml/b.w.). Lesion detection: the brain and spine MRI images of each patient were examined by an expert, board-certified neuro-radiologist and compared with a brain and spine MRI recorded 12 months before, taken under identical conditions, with the same MRI instrument with identical sequences and with the same GD dose. The following parameters were considered: i) the eventual T2-lesion progression in comparison with the previous MRI analysis, ii) the presence of GD-enhanced lesions. The following MRI score was used to evaluate disease status: 1: unchanged, 2: increased lesion load without GD enhancement, 3: presence of GD enhancement.

### Statistical Analysis

The non-parametric Mann-Whitney test was applied to compare quantitative data obtained for healthy and MS patients; in addition, the Kruskal-Wallis and Dunn’s tests were used to compare patient data with MRI parameters. The statistical analysis was performed using R version 2.15.0 [9].

## Results

### Synthesis of PAR and H2AX Phosphorylation

PBMCs from all MS and normal subjects were analysed by IIF in order to evaluate the following parameters: i) PAR synthesis, which occurs under stress conditions, ii) the presence of DNA-break induced γH2AX level, iii) PARP-1 expression. Representative results of the analysis are reported in Figure 1A, where it is evident that the healthy donor (C) has a low basal level of PAR, while in the MS patient (P) a high number of cells positive to the 10H antibody (specific for PAR) were visible, suggesting that the lymphocytes from MS patients have an higher level of stress-stimulated PAR than healthy individuals. Remarkably, we detected more cells positive for the phosphorylation of H2AX in MS patients than in healthy individuals (Figure 1A), indicating that PBMCs from MS patients are characterized by a higher level of DNA damage than control subjects.

Quantitative data from IIF analysis were statistically elaborated by the Mann-Whitney test, and illustrated in Figure 1B. PAR values, expressed as median and quartiles, accounted in controls for 37.00 (23.13–64.16) and in patients for 64.56 (46.66–75.10) (U = 60; p = 0.014), showing for the first time a net increase in PAR synthesis in PBMCs from MS patients compared to healthy subjects. PARP-1 expression did not show any significant difference between healthy donors and MS patients, having median values of 61.00 (quartiles: 35.75–76.50) and 62.24 (quartiles: 35.30–84.32), respectively. The unchanged PARP-1 expression is suggestive of the fact that PAR values are not correlated with the expression level of the enzyme responsible for PAR production (data not shown).

The statistical analysis of γH2AX revealed median values of 25.6 (quartiles: 12.23–35.53) for healthy donors and 35.14 (quartiles: 30.40–68.18) for patients (U = 65; p = 0.025), indicating that for this parameter, the difference between MS patients and normal individuals is significant (Figure 1B).

### Apoptosis in Lymphocytes

We monitored apoptosis in lymphocytes (PBMCs) from patients and healthy individuals by Flow Cytometry (FC), considering two hallmarks, *i.e.* the phosphatidylserine exposure on cell membrane (bound by the Annexin V probe and displayed as green fluorescence by FITC) and/or the most severe damage in the membrane structure rendering it permeable to Propidium Iodide (PI), which intercalates DNA and confers red fluorescence to the cell nucleus of heavily damaged cells. The combination of these two fluorescence emissions allowed discriminate different subpopulations, *i.e.* alive (“non fluorescent”Q3), early apoptotic (only “green fluorescent” Q4), late apoptotic (double “green/red fluorescent” Q2) and necrotic (mostly nude-nuclei only “red fluorescent” Q1) cells. The cell population labeled only with Annexin represents the most reliable fraction undergoing apoptosis, given that the permeability to PI could be suggestive of heavily damaged/necrotic cells. Data relative to early apoptotic cells (green) of all subjects gave rise to graphs shown in Figure 2A and support the evidence that apoptosis level in PBMCs from patients was higher than in healthy subjects: median 1.70 (quartiles: 1.36–2.73) for normal individuals and 3.69 (2.64–5.67) for patients; Mann-Whitney test: U = 47; p = 0.009.

### Correlation between Biological and Clinical Data

The levels of γH2AX in patients were related to different disease status, as assessed by MRI (class 1: unchanged: class 2: increased lesion load without GD enhancement; class 3: presence of GD enchancement). The Kruskal-Wallis test showed a significant variation of γH2AX levels in different MRI classes (p = 0.029); however, the Dunn’s post test demonstrated that only the comparison between γH2AX levels in class 1 and 3 was significant (p = 0.015) (Figure 3).

## Discussion

In MS, the search for biomarkers is particularly relevant for diagnostic and prognostic purposes, to tailor treatments at the individual level and, not least important, to better understand the complex interplay between the various pathogenetic mechanisms. In this respect, we started a pilot study to search for peripheral biomarkers, based on the hypothesis that MS lymphocytes could have altered basal levels of stress and damage, which could possibly be linked to the stage of the disease.

The use of Indirect ImmunoFluorescence and Flow Cytometry according to validated assays provided us with a reliable method to perform a semi-quantitative analysis of stress markers. We focused on the phosphorylation of the histone H2AX (γH2AX), which is a marker of cellular stress [10]; under DSB conditions, γH2AX determines the recruitment of repair factors at the lesion sites and promotes chromatin reorganization [6], [11]. Another stress marker is poly(ADP-ribose) (PAR), synthesized in large quantities by PARP enzymes, whose activity increases when cells are subjected to damage [12]. Both γH2AX and PAR are promising biomarkers potentially useful to monitor the clinical status of different diseases [13]. Moreover, we investigated apoptosis, a physiologically programmed cell death, which guarantees cellular homeostasis and eliminates cells damaged by stress or DNA breakage [14].

Our pilot study was conducted on PBMCs isolated from 19 relapsing-remitting (RR), treatment-naïve MS patients, and 13 healthy volunteers. The analysis was based on immunocytochemistry experiments for PAR synthesis, γH2AX and PARP-1 expression, and on flow cytometry to detect apoptosis. We revealed increased PAR synthesis in PBMCs of MS patients compared to healthy donors; this observation extends to human peripheral lymphocytes the finding of PARP-1 activation observed by histochemistry in a primate model of MS in CNS cells, as well as in infiltrating peripheral blood cells [15]. PARP activation leads to overproduction of PAR, which represents a mediator of neuronal death under stress conditions [16], [17]. In fact, it has been reported that PARP pharmacological inhibition improves neuronal survival in a number of CNS diseases, including MS [18], [19].

With respect to the phosphorylation of H2AX, we detected increased levels of γH2AX in PBMCs of MS patients compared to healthy subjects. To our knowledge, this is an original observation. Given that the phosphorylation of H2AX occurs in the presence of DSBs, it is tempting to correlate these data to an intrinsic persistence of unrepaired cellular damage in MS subjects. Remarkably, when patients were further subdivided in three groups, according to the MRI-based classification of disease phase, we found a significant increase of γH2AX levels between the two most extreme classes of MS aggressiveness (class 1 *vs.* class 3).

Finally, flow cytometry analysis of apoptosis provided promising results. In fact, we found an increased number of lymphocytes with apoptotic hallmarks in samples from MS individuals, in line with the observation of Prieto et al. [5] who reported an enhanced spontaneous apoptosis in MS patients. This evidence supports the hypothesis that apoptosis is an important feature in the pathogenesis of MS [20].

On the whole, our results indicate that PBMC stress biomarkers and apoptotic cell number are increased in RRMS. The biological meaning of our data will be investigated in further experiments, to extend the analysis to other patients and to consider additional markers of apoptosis and cellular stress. Moreover, the association between stress biomarkers and disease activity as measured by MRI could suggest a possible role as “state” markers of MS.

## References

[1] Goverman JM (2011) Immune tolerance in multiple sclerosis. Immunol Rev 241: 228–240. 21488900 10.1111/j.1600-065X.2011.01016.x PMC3088997

[2] McFarland HF, Martin R (2007) Multiple sclerosis: a complicated picture of autoimmunity. Nat Immunol 8: 913–919. 17712344 10.1038/ni1507

[3] Stadelmann C (2011) Multiple sclerosis as a neurodegenerative disease: pathology, mechanisms and therapeutic implications Curr Opin Neurol. 24: 224–229. 10.1097/WCO.0b013e328346056f 21455066

[4] Gonsette RE (2008) Oxidative stress and excitotoxicity: a therapeutic issue in multiple sclerosis? Mult Scler 14: 22–34. 17881394 10.1177/1352458507080111

[5] Prieto A, Díaz D, Barcenilla H, Castrillo C, Monserrat J, et al (2006) Increased spontaneous ex vivo apoptosis and subset alterations in peripheral blood T cells from patients with multiple sclerosis. J Clin Immunol 26: 101–112. 16758338 10.1007/s10875-006-9007-5

[6] Fernandez-Capetillo O, Chen HT, Celeste A, Ward I, Romanienko PJ, et al (2002) DNA damage-induced G2-M checkpoint activation by histone H2AX and 53BP1. Nat Cell Biol 4: 993–997. 12447390 10.1038/ncb884

[7] Shechter D, Costanzo V, Gautier J (2004) ATR and ATM regulate the timing of DNA replication origin firing. Nat Cell Biol 6: 648–655. 15220931 10.1038/ncb1145

[8] Giansanti V, Donà F, Tillhon M, Scovassi AI (2010) PARP inhibitors: new tools to protect from inflammation. Biochem Pharmacol 80: 1869–1877. 20417190 10.1016/j.bcp.2010.04.022

[9] R Development Core (2011) A Language and Environment for Statistical. R Foundation for Statistical Computing, Vienna, Austria.

[10] Mah LJ, El-Osta A, Karagiannis TC (2010) GammaH2AX: a sensitive molecular marker of DNA damage and repair. Leukemia 24: 679–686. 20130602 10.1038/leu.2010.6

[11] Lowndes NF, Toh GW (2005) DNA repair: the importance of phosphorylating histone H2AX. Curr Biol 15: R99–R102. 15694301 10.1016/j.cub.2005.01.029

[12] Tong WM, Cortes U, Wang ZQ (2001) Poly(ADP-ribose) polymerase: a guardian angel protecting the genome and suppressing tumorigenesis. Biochim Biophys Acta 1552: 27–37. 11781113 10.1016/s0304-419x(01)00035-x

[13] Redon CE, Nakamura AJ, Zhang YW, Ji JJ, Bonner WM, et al (2010) Histone gammaH2AX and poly(ADP-ribose) as clinical pharmacodynamic biomarkers. Clin Cancer Res 16: 4532–4542. 20823146 10.1158/1078-0432.CCR-10-0523 PMC2940983

[14] Scovassi AI, Diederich M (2004) Modulation of poly(ADP-ribosylation) in apoptotic cells. Biochem Pharmacol 68: 1041–1047. 15313399 10.1016/j.bcp.2004.04.023

[15] Kauppinen TM, Suh SW, Genain CP, Swanson RA (2005) Poly (ADP-Ribose) Polymerase-1 activation in a primate model of Multiple Sclerosis. J Neurosci Res 81: 190–198. 15931673 10.1002/jnr.20525

[16] Kauppinen TM, Swanson RA (2007) The role of poly(ADP-ribose) polymerase-1 in CNS disease. Neuroscience 145: 1267–1272. 17084037 10.1016/j.neuroscience.2006.09.034

[17] Pemberty WT, Tsunoda I (2009) The importance of NAD in Multiple Sclerosis. Curr Pharm Des 15: 64–99. 19149604 10.2174/138161209787185751 PMC2651433

[18] Alano CC, Garnier P, Ying W, Higashi Y, Kauppinen TM, et al (2010) NAD+ depletion is necessary and sufficient for poly(ADP-ribose) polymerase-1-mediated neuronal death. J Neurosci 30: 2967–2978. 20181594 10.1523/JNEUROSCI.5552-09.2010 PMC2864043

[19] Cavone L, Chiarugi A (2012) Targeting poly(ADP-ribose) polymerase-1 as a promising approach for immunomodulation in multiple sclerosis? Trends Mol Med 18: 92–100. 22078487 10.1016/j.molmed.2011.10.002

[20] Zipp F (2000) Apoptosis in multiple sclerosis. Cell Tissue Res 301: 163–171. 10928289 10.1007/s004410000179

