# Correction: Search for Cellular Stress Biomarkers in Lymphocytes from Patients with Multiple Sclerosis: A Pilot Study

**DOI:** 10.1371/annotation/8c8710a2-bd43-4f65-b21e-69ec522c4f22

**Published:** 2012-11-01

**Authors:** Sabrina Grecchi, Giuliano Mazzini, Antonella Lisa, Marie-Therese Armentero, Roberto Bergamaschi, Alfredo Romani, Fabio Blandini, Carol Di Perri, Anna Ivana Scovassi

The following information was missing from the Funding section: AIS laboratory is partially funded by the SepiAs project from the Italian Ministry of Health. AL is supported by the CARIPLO grant n. 2010-0253.

